# MODY2 in Asia: analysis of GCK mutations and clinical characteristics

**DOI:** 10.1530/EC-20-0074

**Published:** 2020-05-06

**Authors:** Yuan Zhou, ShengNan Wang, Jing Wu, JianJun Dong, Lin Liao

**Affiliations:** 1 Department of Endocrinology and Metabology, The First Affiliated Hospital of Shandong First Medical University, Ji-nan, China; 2 Laboratory of Endocrinology, Medical Research Center, Shandong Provincial Qianfoshan Hospital, The First Affiliated Hospital of Shandong First Medical University, Ji-nan, China; 3 Department of Endocrinology, Qilu Hospital of Shandong University, Ji-nan, China; 4 Department of Endocrinology and Metabology, Shandong Provincial Qianfoshan Hospital, Cheeloo College of Medicine, Shandong University, Ji-nan, China

**Keywords:** MODY2, GCK, Asian, characteristics

## Abstract

**Aims:**

Heterozygous inactivating mutations in the GCK gene cause the familial, mild fasting hyperglycaemia named MODY2. Many patients with MODY2 in Asia have delayed timely treatment because they did not receive the correct diagnosis. This study aims to analyze the clinical characteristics and GCK mutations in Asian MODY2.

**Methods:**

We have collected 110 Asian patients with MODY2 from the PubMed, Embase, Medline, Web of Science, CNKI, and Wanfang with the following search terms: ‘maturity-onset diabetes of the young’ OR ‘MODY’ OR ‘maturity-onset diabetes of the young type 2’ OR ‘MODY2’ OR ‘GCK-DM’ OR ‘GCK-MODY’. Both mutations of GCK and clinical characteristics of MODY2 were analyzed.

**Results:**

There were 96 different mutations that occurred in coding regions and non-coding regions. Exon 5 and 7 were the most common location in coding regions and missense was the primary mutation type. The proportion of probands younger than 25 was 81.8%, and 81.4% of the probands had family history of hyperglycaemia. Ninety percent and 93% of Asian MODY2 probands exhibited mild elevation in FPG (5.4–8.3 mmol/L) and HbA1c (5.6–7.6%), respectively.

**Conclusions:**

In most Asian patients, MODY2 occurred due to GCK mutation in coding regions, and exon 5 and 7 were the most common locations. FPG, HbA1c, and familial diabetes were important reference indicators for diagnosing MODY2. Altogether, the study indicates that for the young onset of diabetes with mild elevated blood glucose and HbA1c and family history of hyperglycaemia, molecular genetic testing is suggested in order to differentiate MODY2 from other types of diabetes earlier.

## Introduction

Currently, diabetes has become a public health problem that has garnered worldwide attention. In addition to the well-known type 1 diabetes (T1D) and type 2 diabetes (T2D), a growing number of special types of diabetes and their gene mutations have been discovered with continuous research.

MODY (maturity-onset diabetes of the young) refers to a heterogeneous group of monogenic forms of diabetes caused primarily by insulin secretion defects (1). It was first described as a single clinical entity in a large family in 1960, and generation familial history suggested that MODY was an early onset mild diabetes (usually before age 25), autosomal dominant inheritance and predominance of insulin deficiency (2). Since the breakthroughs of molecular genetic testing technology from 1990s, relevant studies have recognized that MODY comprises several different clinical syndromes of familial diabetes resulting from specific molecular defects (3). There are 14 genes that have been proven related to MODY, including HNF1A, GCK, HNF4A, HNF1B, ABCC8, and so on (4). In Europe, MODY accounted for 1–2% of the total diabetes population (5), but the exact prevalence of MODY all over the world was not known. Moreover, it was estimated to be responsible for 2 to 5% of cases of non-insulin-dependent diabetes mellitus (6).

Glucokinase (GCK, also named hexokinase IV) belongs to the hexokinase family and plays critical roles in glucose homeostasis (7, 8). The GCK enzyme constitutively expresses and catalyzes the initial rate limiting step in the glycolytic pathway by ATP-dependent phosphorylation of glucose to glucose-6-phosphate in presence of Mg ions (7). Heterozygous inactivating mutations in the GCK gene cause the familial, mild fasting hyperglycaemia named MODY2 (9). Clinical features of MODY2 include a non-progressive slight increase in glycated hemoglobin (HbA1c), usually between 5.6% and 7.6%, and mildly raised fasting glucose (usually between 5.4–8.3 mmol/L) (10). The current strategy for identifying patients with a potential MODY2 mutation is to combine the clinical characteristics and molecular genetic testing (11, 12, 13).

The correct diagnosis is especially critical for patients with MODY2, because MODY2 patients do not require antihyperglycemic therapy except sometimes during pregnancy (14), and multiple studies have shown that no complications ensue in the absence of glucose-lowering therapy (15). Due to insufficient knowledge of MODY2, it was often misclassified as T1D or T2D and the patients were often treated improperly (16, 17). In this article, we analyzed the clinical characteristics and GCK mutations of Asian MODY2 patients, in order to facilitate the screening and diagnosis of MODY2 in Asia.

## Subjects and methods

PubMed, Embase, Medline, Web of Science, the China National Knowledge Infrastructure (CNKI), and Wanfang were searched from the date of their inception to June 30, 2019 without language restrictions. The search strategy was composed of the following search terms: ‘maturity-onset diabetes of the young’ OR ‘MODY’ OR ‘maturity-onset diabetes of the young type 2’ OR ‘MODY2’ OR ‘GCK-DM’ OR ‘GCK-MODY’. All the enrolled studies confirmed with the following criteria: (1) articles aimed at Asian population; (2) the detailed clinical data of probands should have at least accurate FPG; and (3) described the GCK mutations and the patients were confirmed as MODY2 by DNA test. The flow chart showed identification of MODY2 in Asian countries and the reasons for their exclusions (Supplementary Fig. 1, see section on supplementary materials given at the end of this article). The definition of Asia is the continent that is to the east of Europe, the west of the Pacific Ocean, and the north of the Indian Ocean.

The following clinical and laboratory variables were studied: (1) country; (2) gender; (3) age at diagnosis; (4) familial history; (5) diabetes therapy (oral hypoglycemic agents (OHA), insulin, and diet); (6) BMI at recruitment; (7) laboratory test results at diagnosis, including fasting plasma glucose (FPG), 2-h postprandial plasma glucose (2-h PG), fasting insulin (Fins), 2-h postprandial insulin (2-h Ins), hemoglobin A1c (HbA1c), total cholesterol (TC), triglyceride (TG), high-density lipoprotein cholesterol (HDL-c), and low-density lipoprotein cholesterol (LDL-c). Amino acid substitution and type and position of mutations in the respective gene were recorded. Methods used in the molecular diagnosis of published cases had to be described in detail (see references at Table 1).

**Table 1:** The detailed information of Asian MODY2 studies.

Country	Enrolled articles	Patients	Reference
China	15	48	(18, 19, 20, 21, 22, 23, 24, 25, 26, 27, 28, 29, 30, 31, 32)
Japan	2	38	(33, 34)
Iran	1	1	(35)
Turkey	2	19	(36, 37)
Korea	3	4	(38, 39, 40)

## Results

### Gene mutations in MODY2

Twenty-three publications describing MODY2 mutations in Asian subjects were found using the aforementioned search terms. These publications dealt with 110 individuals from different families with a clinical profile consistent with MODY2. Of note, all the selected patients were the only proband in the families. They came from unrelated families from Asian countries and were born from non-consanguineous parents. The studies were collected from 5 countries in Asia, 15 of which were in China, accounting for the majority. The number of patients in Japan followed closely behind. The detailed information of enrolled countries and individuals were described in Table 1.

We recorded GCK mutations on each patient such as position of mutation and amino acid substitution. Methods used in the molecular diagnosis were described in detail in the original publications (Table 2). In total, 90 different GCK mutations were identified in coding regions, which included 76 (84.4%) missense, 4 (4.5%) nonsense, 6 (6.7%) deletions, 3 (3.3%) duplicates, and (1.1%) all exon deletion. All of these mutations were predicted to be deleterious, analyzed by online bioinformatics tools. Another six mutations were located outside the coding region involving c.1254-1G > C, c.208 + 3A > T, c.46-2A > G, c.483 + 2 T > A, c.679 + 1G > A, and IVS1B+12A > T. It is worth noting that c.46-2A > G was found in two unrelated families in Turkey, and four types of missense mutations (c.571C>T, c.572G>A, c.617C>G, and c.661 G>A) were found in five, three, three, and two unrelated families, respectively. Moreover, GCK mutations were distributed throughout the exon 1–10. Of note, the largest proportion (20.9%, 23/110) of the mutations was in exon 5, and 17 probands had the mutations in exon 7.

**Table 2:** GCK mutations of Asian MODY2 patients.

Country	No.	Exon	cDNA	Protein
China	1	7	c.749 G > A	p.R250H
	2	2	c.127 C > T	p.R43C
	3	7	c.781 G > C	p.G261R
	4	7	c.661 G > A	p.R221K
	5	7	c.771 G > A	p.W257STOP
	6	5	c.571 C > T	p.R191W
	7	5	c.507 G > C	p.K169N
	8	5	c.502 A > G	p.T168A
	9	7	c.704 T > A	p.M235T
	10	5,9	c.1136C > A + c.571C > T	p.A379E+p.R191W
	11	6	c.645 C > A	p.Y215STOP
	12	1	c.34 - 4 + 15del26	NA
	13	5	c.544 G > A	p.V182M
	14	Intronic 4	c.483 + 2 T > A	
	15	9	c.1121_1132del12	p.V374_A377del
	16	4	c.451_453delTCC	p.S151del
	17	Intronic 6	c.679 + 1G > A	
	18	2	c.169_170delATinsG	p. M57GfsX29
	19	8	c.883 G > A	p.G295S
	20	5	c.572 G > A	p.R191E
	21	2	c.122 T > C	P.M41T
	22	6	c.661 G > A	p.E221K
	23	7	c.771 G > A	p.W257ter
	24	1	c.13 G > C	p.V5L
	25	7	c.1174 G > T	p.M391R
	26	9	c.1190 G > T	p.R397L
	27	5	c.485 G > A	p.G162D
	28	2	c.128 G > A	p.R43H
	29	6	c.676 G > A	p.V226M
	30	10	c. 1348 G > T	p.A450T
	31	3	NA	p.T82P
	32	9	NA	p.R377L
	33	2	NA	p.G44S
	34	7	NA	p.A259S
	35	2	NA	p.R43H
	36	7	NA	p.R250C
	37	2	NA	p.G44S
	38	9	NA	p.T354M
	39	4	NA	p.D135E
	40	9	NA	p.T354M
	41	4	NA	p.D135E
	42	8	NA	p.G318R
	43	5	c.556 C > T	p.R186 stop
	44	4	c.367-374dupTTCGACTA	p.Ile126fs
	45	5	c.571 C > T	p.R191W
	46	6	c.626 C > T	p.T209M
	47	7	c.824 G > A	p.R275H
	48	1	IVS1B+12 A > T	
Iran	49	8	c.1010delA	p.*352stop
Turkey	50	2	c.151 G > T	p.E51*
	51	10	c.1396 T > A	p.*466R
	52	9	c.1148 C > T	p.S383L
	53	Intronic	c.46-2 A > G	
	54	3	c.214 G > A	p.G72R
	55	5	c.508 G > A	p.G170S
	56	4	c.368 T > C	p.F123S
	57	7	c.823 C > T	p.R275C
	58	2	c.173 T > C	p.L58P
	59	5	c.572 G > A	p.R191Q
	60	Intronic	c.46-2 A > G	
	61	Intronic	c.208 + 3 A > T	
	62	9	c.1178 T > C	p.M393T
	63	7	c.737 G > C	p.G246A
	64	10	c.1256 T > G	p.F419C
	65	Intronic	c.1254-1 G > C	
	66	3	c.452 C > G	p.S151C
	67	9	c.1099 G > A	p.V367M
	68	5	c.506 A > G	p.K169R
Japan	69		All exon deletion	
	70	7	c.706G > A	p.E236K
	71	6	c.617C > T	p.T206M
	72		All exon deletion	
	73	7	c.781 G > A	p.G261R
	74	2	c.118 G > A	p.E40K
	75	2	c.175 C > T	p.P59S
	76	4	c.364 C > T	p.L122F
	77	5	c.572 G > A	p.R191Q
	78	6	c.617 C > T	p.T206M
	79	5	c.577 G > T	p.G193W
	80	10	c.1278_1286dup	p.S426_R428dup
	81	5	c.571 C > T	p.R191W
	82	5	c.538 A > C	p.N180H
	83	9	c.1232 C > T	p.S411F
	84	6	c.617 C > G	p.T206R
	85	4	c.437 T > G	p.L146R
	86	2	c.76 C > T	p.Q26*
	87	8	c.1019 G > C	p.S340T
	88	8	c.895 G > C	p.G299R
	89	7	c.751 A > G	p.M251V
	90	9	c.1055 T > G	p.L352R
	91	8	c.898 G > T	p.E300*
	92	5	c.571 C > T	p.R191W
	93	7	c.835_836 del	p.E279Efs*49
	94	5	c.571C > T	p.R191W
	95	9	c.1144-1149 dup	p.C382_S383dup
	96	5	c.556 C > T	p.R186*
	97	3	c.234 C > G	p.D78E
	98	7	c.764 C > G	p.T255S
	99	9	c.1142 T > G	p.M381R
	100	6	c.1517 C > T	p.T206M
	101	2	c.130 G > A	p.G44S
	102	5	c.575 G > A	p.R191Q
	103	2	c.182 A > G	p.Y61C
	104		All exon deletion	
	105	5	c.576 G > T	p.G193W
	106	5	c.500 G > A	p.W167X
Korea	107	9	c.1257-20_1315del	NA
	108	2	c.92 T > C	p.L30P
	109	9	c.1151 C > T	p.S383P
	110	5	c.191 C > T	p.R191W

### Clinical characteristics of MODY2

**Figure 1:**
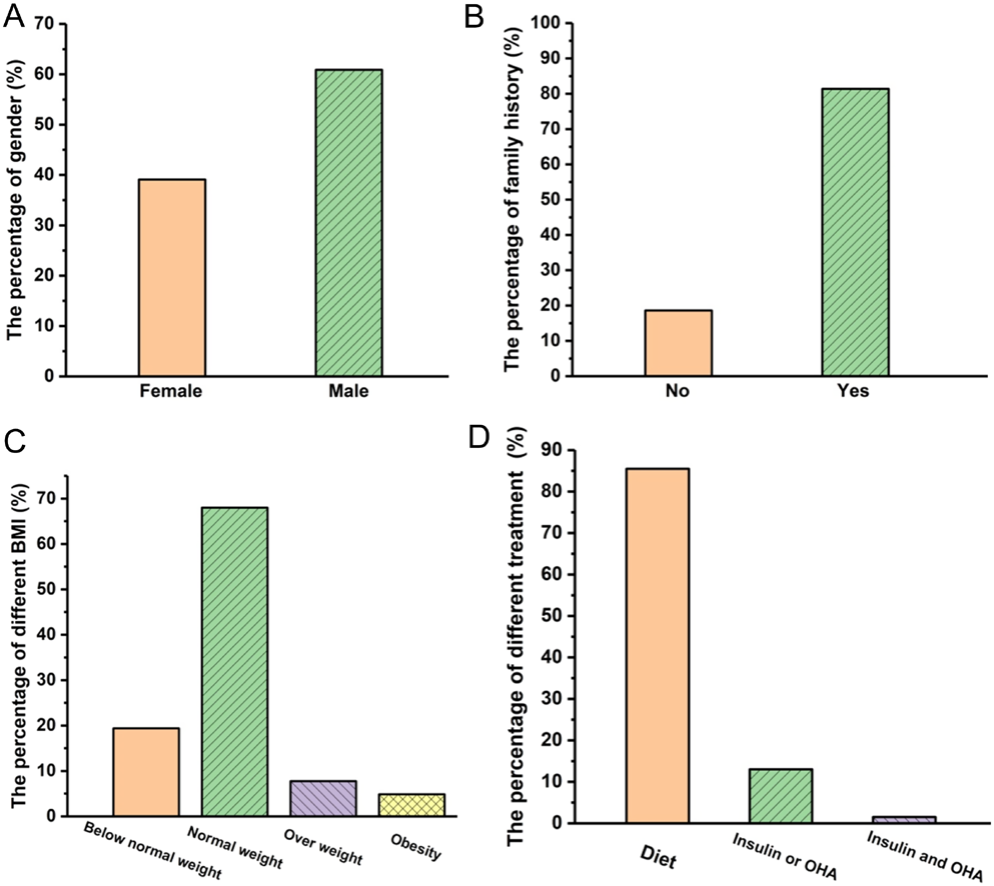
Clinical characteristics of Asian MODY2 patients. (A, B, C, and D) The proportion of several clinical characteristics in enrolled probands: (A) gender, (B) familial history of hyperglycaemia, (C) BMI, and (D) treatment before diagnosis.

The gender information of all 110 patients are available in Fig. 1A, of which 67 (60.9%) are male. In 70 families, 57 (81.4%) had a family history of hyperglycaemia (Fig. 1B). BMI or BMI percentiles at diagnosis were available for 103 of the 110 probands. For patients with BMI percentile data, we classified them according to WHO and CDC standards (41). For patients with BMI data, we classified them according to WHO standard (42). The distribution of BMI in Asian MODY2 patients are shown in Fig. 1C. Seventy of them (68%) had normal body weight, which was different from the T1D (low body weight is dominant) or T2D (over body weight is dominant). The group of below normal weight, overweight, and obesity accounted for 19.4%, 7.8%, and 4.8%, respectively. The treatments of 69 probands were provided in the original articles (Fig. 1D) and we found that 59 (85.5%) patients underwent diet therapy before DNA diagnosis. Whereas, nine (13%) probands received insulin or oral hypoglycaemic agents (OHA) and one person (1.5%) was treated with insulin and OHA.

**Figure 2:**
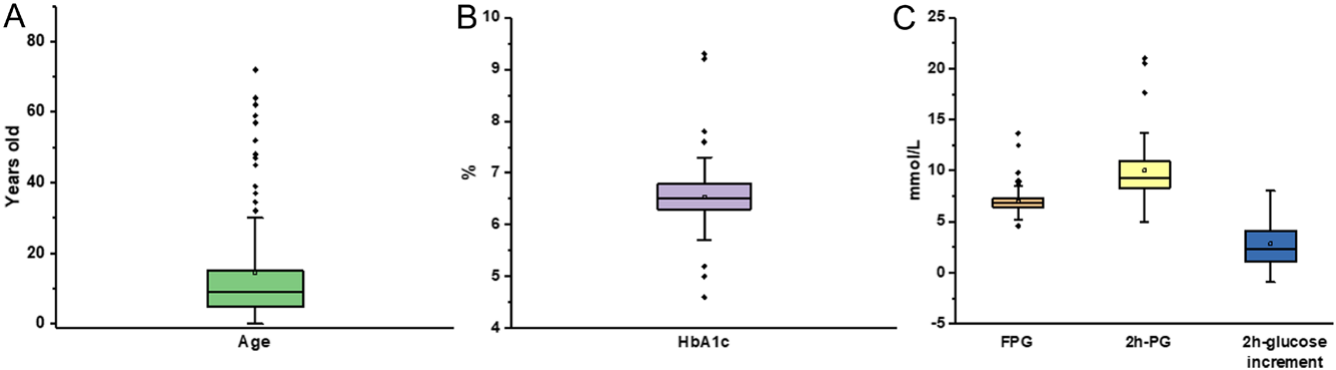
Whisker plot for continuous clinical data of Asian MODY2 patients. (A, B, and C) Continuous data for the variables of (A) age, (B) HbA1c, (C) FPG, 2h-PG, and 2h-glucose increment.

The clinical data at diagnosis of MODY2 patients are shown in Fig. 2 and Table 3. The age information of all 110 patients is shown in Fig. 2A, and most of the probands (81.8%) were under 25 years of age. HbA1c data (Fig. 2B) were available for 100 of the 110 individuals. HbA1c ranged between 4.6 and 9.3%, and the average HbA1c levels were 6.54 ± 0.65%. The probands with HbA1c between 5.6 and 7.6% account for 93% (93/100). FPG, 2h-PG and 2h-glucose increment were available for 110, 62, and 62 probands, respectively (Fig. 2C). The FPG ranged between 4.55 and 13.66 mmol/L, the value was 6.98 ± 1.17 mmol/L (mean ± s.d.). Ninety percent (99/110) of the probands had the levels of FPG within the range of 5.40–8.30 mmol/L. The levels of 2h-glucose increment in 32 individuals (51.6%) were below <3.00 mmol/L, the levels of 2h-PG in 47 (75.8%) probands were below 11.10 mmol/L. TC and TG in 26 patients and LDL and HDL in 25 patients were also analyzed; however, no significant difference was found.

**Table 3:** Clinical data of Asian MODY2 patients (probands only).

Subjects	No. of patients	Mean ± s.d.
FPG (mmol/L)	110	6.98 ± 1.17
2h-PG (mmol/L)	62	10 ± 2.86
2h-glucose increment (mmol/L)	62	2.82 ± 2.03
HbA1c (%)	100	6.54 ± 0.65
Fins (mIU/L)	78	10.17 ± 11.19
2-h Ins (mIU/L)	19	35.16 ± 23.82
TC (mmol/L)	26	4.46 ± 0.88
TG (mmol/L)	26	1.1 ± 0.84
LDL	25	2.39 ± 0.62
HDL	25	1.26 ± 0.37

## Discussion

Most studies of MODY2 were conducted in European Caucasians (43). However, the studies of MODY2 in Asia were not much. The reasons for low MODY2 diagnosis might be the following two. First, most of the MODY2 patients do not have obvious symptoms nor signs. Second, some of MODY2 patients are misdiagnosed as having type 1 or type 2 diabetes or impaired fasting glucose (5). Previous reports have shown that MODY2 was the most common form of MODY both in China and Japan when asymptomatic patients were systematically screened (44, 45).

Although one of the characteristics of MODY2 was insidious onset, 81.8% of the MODY2 probands enrolled in our study were under 25 years of age at first diagnosis. Laboratory tests are helpful for early identification of MODY2 patients. Most MODY2 patients exhibited mild elevation in FPG levels and about 90% of patients had FPG values between 5.40–8.30 mmol/L. However, the majority of MODY2 patients did not have postprandial hyperglycemia (75.8% of patients had a level of 2h-PG below 11.10 mmol/L), and the increments of blood glucose were 2.82 ± 2.03 mmol/L during oral glucose tolerance test. Study showed that most of GCK mutations altered the set-point of insulin secretion and that their pancreas could still could secret insulin (46). So, patients with MODY2 do not need hypoglycemic agents treatment (14).

There were 11 MODY2 probands that did not meet the diagnostic criteria of HbA1c (5.6–7.6%) nor FPG (5.40–8.30 mmol/L). The details are as follows: (1) The FPG of one proband (34) was 4.55 mmol/L, and HbA1c was not available. The proband was diagnosed as MODY2 in the original article because of a harmful mutation (c.1517 C > T); (2) The FPG of five probands were 4.60 (33), 8.50 (30), 8.71 (37), 8.90 (23), 8.99 (36) mmol/L, respectively, and it did not meet the criteria of FPG. However, their HbA1c were between 5.6 and 7.6%. All of them were identified mutations and diagnosed as MODY2 in original articles; (3) One proband (18) had FPG 5.20 mmol/L and HbA1c 5.0%; however, the original article mentioned that both the proband and his father had mild raise in glucose. Moreover, both of them had harmful mutations (c.749 G > A); (4) One proband (22) had FPG 12.48 mmol/L and the HbA1c was non-available. The proband and her father were both detected with c.13 G > C mutation. Unfortunately, the original article did not mention whether the proband coexisted with other type of diabetes; (5) One 45-year-old proband (31) with FPG 5.27 mmol/L and HbA1c 9.3%. The proband was injected insulin when he was diagnosed as MODY2; (6) There were two untreated probands (31) (59 and 48 years old) with FPGs 9.80 mmol/L and 13.66 mmol/L and HbA1c 7.8% and 9.2%, respectively. The original article mentioned that the probands in (5) and (6) did not follow the criteria because these probands had a co-existing diagnosis of type 2 diabetes.

Appropriate sequencing method should be cost-effective in order to early diagnose MODY2. The methods of gene screening in enrolled studies included Sanger sequencing, targeted next generation sequencing (NGS) panels, and multiplex ligation-dependent probe amplification (MLPA). Both Sanger sequencing and NGS could detect the potentially causative small nucleotide polymorphisms (SNPs), small insertions/deletions, frameshift mutations, and null mutations (47). They are the common methods in diagnosis of MODY2. MLPA is typically applied to detect large deletion in genes. It is often used where a diagnosis of MODY2 is strongly suspected but no mutation is found by DNA sequencing (48).

Our study has several limitations. First, in order to analyze the characteristics of the MODY2 patients in Asia, the studies without adequate information were excluded. All the probands involved in our article had at least FPG, and patients were diagnosed as MODY2 by DNA tests. We regret that not all Asian countries were shown in the results, such as India which was completely missed. Second, we found that the most common mutations were located at exons 5 and 7 in Asian MODY2 patients. We still need further studies for the purpose of explaining the more precise molecular mechanism of MODY2.

In summary, our study showed that 90% (99/110) and 93% (93/100) of Asian MODY2 probands exhibited mild elevation in FPG (5.4–8.3 mmol/L) and HbA1c (5.6–7.6%). Most probands (81.4%, 57/70) had a family history of hyperglycaemia. We found that 79.2% (76/96) of GCK mutations in Asian patients were missense. We also found that 93.8% (90/96) of GCK mutations were located in the coding region and unevenly distributed along the 10 exons of the gene and that exon 5 (20.9%, 23/110) and exon 7 (15.5%, 17/110) were the most common locations. Altogether, the study indicates that for the young onset of diabetes with mild elevated blood glucose and HbA1c and family history of hyperglycaemia, molecular genetic testing is suggested in order to differentiate MODY2 from other types of diabetes earlier.

## Supplementary Material

Fig S1. Flowchart of the systematic search process.

## Declaration of interest

The authors declare that there is no conflict of interest that could be perceived as prejudicing the impartiality of the research reported.

## Funding

This work was funded by National Natural Science Foundation of China (81670757, 81570742) and the Grant for the Development of Science and Technology of Ji-nan City (201602172).

## Author contribution statement

J Dong and L Liao contributed equally to this work.
