# Supplementary material for: MODY2 in Asia: analysis of GCK mutations and clinical characteristics
Source: Endocr Connect. 2020 May 6;9(5):471–8. doi: 10.1530/EC-20-0074 (PMC7274558; doi:10.1530/EC-20-0074)
Supplement: Fig S1. Flowchart of the systematic search process. [file supplementary_figure_1.pdf]

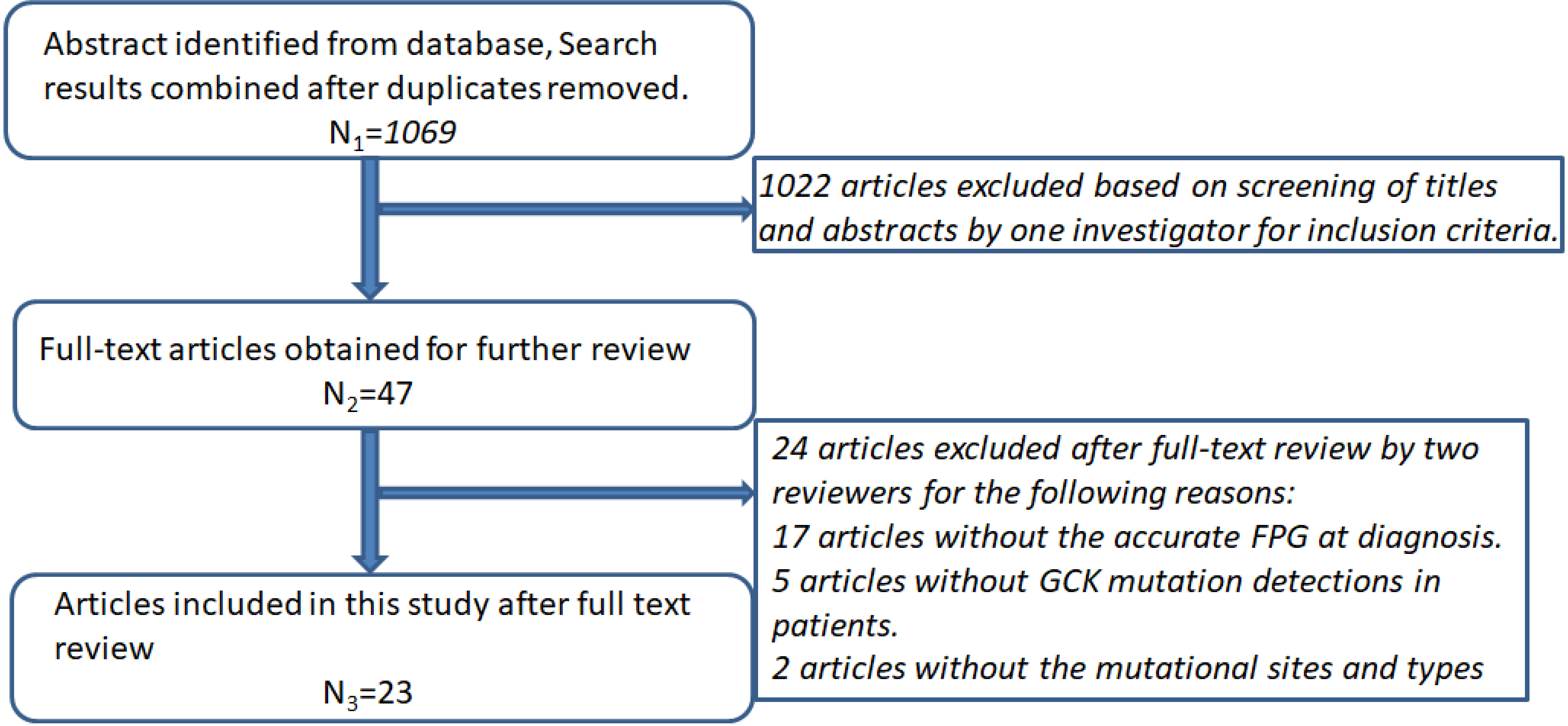
Abstract identified from database, Search results combined after duplicates removed.
N1=1069
1022 articles excluded based on screening of titles and abstracts by one investigator for inclusion criteria.
Full-text articles obtained for further review
N2=47
24 articles excluded after full-text review by two reviewers for the following reasons:
17 articles without the accurate FPG at diagnosis.
5 articles without GCK mutation detections in patients.
2 articles without the mutational sites and types
Articles included in this study after full text review
N3=23
